# The Effect of Radiation and Chemoradiation Therapy on the Head and Neck Mucosal Microbiome: A Review

**DOI:** 10.3389/fonc.2021.784457

**Published:** 2021-12-02

**Authors:** Ivry Zagury-Orly, Nader Khaouam, Jonathan Noujaim, Martin Y. Desrosiers, Anastasios Maniakas

**Affiliations:** ^1^ Faculty of Medicine, Université de Montréal, Montreal, QC, Canada; ^2^ Department of Radiation Oncology, Hôpital Maisonneuve-Rosemont, Montreal, QC, Canada; ^3^ Department of Oncology, Hôpital Maisonneuve-Rosemont, Montreal, QC, Canada; ^4^ Centre de Recherche du Centre Hospitalier de l’Université de Montréal, Montreal, QC, Canada; ^5^ Division of Otolaryngology-Head and Neck Surgery, Centre Hospitalier de l’Université de Montréal, Montreal, QC, Canada; ^6^ Division of Otolaryngology-Head and Neck Surgery, Hôpital Maisonneuve-Rosemont, Montreal, QC, Canada; ^7^ Department of Experimental Surgery, McGill University, Montreal, QC, Canada; ^8^ Department of Head and Neck Surgery, The University of Texas MD Anderson Cancer Center, Houston, TX, United States

**Keywords:** head and neck cancer, microbiome, radiation therapy, chemoradiation therapy, side effects

## Abstract

Radiation (RT) and chemoradiation therapy (CRT) play an essential role in head and neck cancer treatment. However, both cause numerous side effects in the oral cavity, paranasal sinuses, and pharynx, having deleterious consequences on patients’ quality of life. Concomitant with significant advances in radiation oncology, much attention has turned to understanding the role of the microbiome in the pathogenesis of treatment-induced tissue toxicity, to ultimately explore microbiome manipulation as a therapeutic intervention. This review sought to discuss current publications investigating the impact of RT and CRT-induced changes on the head and neck microbiome, using culture-independent molecular methods, and propose opportunities for future directions. Based on 13 studies derived from a MEDLINE, EMBASE, and Web of Science search on November 7, 2021, use of molecular methods has uncovered various phyla and genera in the head and neck microbiome, particularly the oral microbiome, not previously known using culture-based methods. However, limited research has investigated the impact of RT/CRT on subsites other than the oral cavity and none of the studies aimed to examine the relationship between the head and neck microbiome and treatment effectiveness. Findings from this review provide helpful insights on our current understanding of treatment-induced oral mucositis, dental plaque, and caries formation and highlight the need for future research to examine the effect of RT/CRT on the sinonasal and oropharyngeal microbiome. In addition, future research should use larger cohorts, examine the impact of the microbiome on treatment response, and study the effect of manipulating the microbiome to overcome therapy resistance.

## Introduction

Head and neck cancers (HNC) are responsible for nearly 330,000 annual deaths worldwide (1). Most HNCs originate from the mucosal epithelium of the upper aerodigestive tract (2). Radiation therapy (RT) is an essential component of HNC treatment. RT alone or combined with chemotherapy [i.e., chemoradiation therapy (CRT)] can be offered as primary treatment, as an adjuvant following surgery, or as palliation for unresectable HNCs (3). The desired tumoricidal effect of RT inadvertently injures normal cells adjacent to the area targeted (4). This results in several side effects, including oral mucositis (OM), xerostomia, dysphagia, odynophagia, and RT-induced chronic rhinosinusitis (CRS), having a significant impact on the quality of life of patients with HNC (3, 5, 6).

In efforts of reducing radiation toxicity, significant advances have occurred in the field of RT (4): (i) emergence of new radiation techniques such as intensity-modulated RT; (ii) improvement in optimizing dose schedules, planning, and simulation using functional imaging; and (iii) combining radiation and systemic agents. Other research focuses on improving our molecular understanding of RT-induced tissue injury (4), in part through study of the human microbiome (7).

In this review, we discuss the current research investigating the impact of RT for HNC on the head and neck mucosal microbiome using culture-independent molecular methods and present opportunities for future directions.

## Microbiome Research: An Overview

### The Basics

The microbiota refers to the collection of microorganisms in a given environment, whereas the microbiome consists of the microbiota and their genes (8). Metataxonomics is the process by which the microbiota is given its classification. It provides information on the distribution and diversity of microorganisms. This process includes DNA extraction from stool sampling or buccal and nasopharyngeal swabs and subsequent molecular analysis of amplified and sequenced genes, most commonly 16S ribosomal RNA (rRNA) genes (for bacteria) and 18S rRNA genes (for eukaryotes) (9, 10). Viruses, although part of the microbiota, are instead detected by next-generation sequencing (NGS) technologies (9, 10), capable of detecting microorganisms at the strain level and analyzing genetic material (metagenomics) that reflect microbiome *function* (8, 9). Other molecular methods, combined with metagenomics, are increasingly recognized for providing a more integrated analysis (11).

The healthy oral cavity primarily consists of bacterial genera—both gram-positive (e.g., *Peptostreptococcus, Streptococcus, Stomatococcus, Actinomyces, Bifidobacterium, Corynebacterium, Lactobacillus, Propionibacterium*) and gram-negative (e.g., *Moraxella, Neisseria, Veillonella, Campylobacter, Capnocytophaga, Eikenella, Fusobacterium, Hemophilus, Leptotrichia, Prevotella, Treponema*)—but also inhabits protozoa (e.g., *Entamoeba gingivalis and Trichomonas tenax*), fungi (most commonly *Candida species*), and viruses (12). A healthy sinonasal microbiome also houses its own set of characteristic genera, namely, *Staphylococcus, Propionibacterium, Corynebacterium, and Moraxella* (13).

### Radiation Therapy and Microbiome Disruption

The human microbiome contains both beneficial and harmful microorganisms. The adequate balance and interaction between both types enable symbiosis—where both the human and microbiota may benefit—predicting a healthy state, whereas perturbed homeostasis characterizes dysbiosis—a microbial community associated with a diseased state (14). Dysbiosis is believed to be the result of multiple influencing factors—intrinsic (e.g., age, sex, psychological stress, and genetics) and extrinsic (e.g., smoking and alcohol consumption, antibiotics, surgery, RT, and CRT) that perturb the host-microbe-environment interactions (11).

Much of what we currently understand on the effects of RT on the human microbiome derives from gut research (15). A bidirectional effect has been described where RT disrupts the microbiome and through its involvement in mediating immune response, the disrupted microbiome subsequently interferes with RT effectiveness (11). Radiation-induced microbiome disruption is thought to result from two principal mechanisms involving inflammation (11, 16). First, radiation directly leads to tissue oxidation and inflammation, which alters the local microenvironment and promotes dysbiosis. Microbiome dysbiosis is thought to disrupt the immune system, leading to an upregulation of pro-inflammatory (e.g., Th17) and downregulation of anti-inflammatory molecules (e.g., regulatory T cells). Second, radiation causes toxic damage to the epithelium, resulting from cellular DNA and RNA damage, that leads to cell death by apoptosis, followed by ulceration, and exposure of tissue not supposed to be in contact with bacteria, characterized by bacterial translocation and colonization, further increasing the inflammatory response.

Finally, bacteriophages, which are viruses that can kill bacteria, are abundant on epithelial surfaces and are thought to play an important role in human tissue homeostasis and maintenance of health (17). However, to date, these have primarily been discussed in the context of tailored antimicrobial therapy (18), and to our knowledge, metagenomic research assessing the impact of RT on bacteriophages is limited. Nonetheless, we hypothesize that if RT causes a loss of these protective phages, commensal strains may begin showing pathogenic features, or pathogenic strains may become dominant. Such pathogenic species may then readily proliferate in the context of an ulcerated and inflammatory irradiated tissue (9, 18).

### Clinical Relevance

The study of microbiome dysbiosis has led to a better understanding of local and systemic disease *occurrence*. Although the oral microbiome only represents a fraction of the human microbiome, largely represented by the gut (19), oral dysbiosis is linked to periodontitis, dental caries, and HNC, but also to endocarditis, atherosclerosis, Alzheimer’s disease, and diabetes (20). Similarly, gut dysbiosis has been associated with gastrointestinal disease occurrence (e.g., inflammatory bowel disease, irritable bowel syndrome, and clostridium difficile infection), as well as neurodegenerative disease, diabetes, rheumatoid arthritis, and obesity (14, 15). The gut microbiome is also linked to HNC; symbiosis leads to favorable outcomes whereas dysbiosis has been associated with the development of psychoneurological symptoms related to cancer treatment (21). Moreover, specific microorganisms may be associated with *refractory* disease. For example, the presence of *Staphylococcus aureus* in the sinonasal microbiome appears to predict failure of endoscopic sinus surgery in patients with CRS at high risk of recurrence (22). Identification of such microorganisms, then allows for clinical trials that measure the effectiveness of specific antibiotic regimens in reducing the abundance of harmful pathogens in patients with refractory disease such as CRS (23).

As we will explore in this review, therapeutic modalities that do not target the microbiome intentionally, such as RT or CRT, cause changes in the microbiota that may perturb an otherwise symbiotic environment and help explain decreased treatment efficacy and undesirable side effects. Understanding *how* and *why* the microbiome is modified in response to RT has led to the emergence of probiotics and fecal microbial transplantation used to recreate an appropriate balance of pro-and anti-inflammatory cells with aims of enhancing response to RT and minimizing toxicity (11, 24).

## Radiation Therapy and Changes in the Head and Neck Microbiome

A search was conducted on the MEDLINE (**Table 1**), EMBASE, and Web of Science databases on November 7, 2021, combining the following concepts: (i) head and neck malignancy, (ii) radiation therapy, and (iii) microbiome, using various keywords. A total of 378 studies were screened, 13 of which were included in our qualitative synthesis (**Table 2**). Studies were excluded if any of the concepts included in the search were not central to the article or if they used culture-based methods.

**Table 1:** MEDLINE Search Strategy.

Concepts	#	Searches	Results
			(November 7, 2021)
A	1	exp otolaryngology/OR exp otorhinolaryngologic surgical procedures/OR exp otorhinolaryngologic diseases/su OR exp ear/su OR exp nose/su OR exp pharynx/su OR “head and neck neoplasms”/su OR parathyroid neoplasms/su OR exp thyroid neoplasms/su OR tracheal neoplasms/su OR exp salivary glands/su OR bronchoscopy/OR thyroidectomy/OR parathyroidectomy/OR glossectomy/	241426
A	2	(“ear nose and throat” OR otorhinolaryngolog* OR otolaryngolog* OR otolog* OR laryngolo* OR rhinolog* OR ent OR otl OR oral OR nasal OR sinus OR sinonasal OR ear).ab,ti	991257
A	3	[(“head and neck” OR laryn* OR pharyn* OR thyroid OR oral OR parathyroid OR trachea* OR sinus OR nasal OR throat) adj3 (cancer* OR neoplasm* OR carcinoma* OR adenocarcinoma*)].ab,ti	146864
	4	or/1-3	1247980
B	5	exp radiotherapy/OR exp radiofrequency therapy/su OR exp chemoradiotherapy/su OR exp radioimmunotherapy/su OR [radi* adj1 (therap*)].ab,ti OR [(radiotherap* OR irradiat* OR postradiation)].ab,ti	552683
C	6	exp microbiota/OR (microbiom*).ab,ti	69893
	7	4 and 5 and 6	58

The asterisk (*) represents any group of characters, including no character.

**Table 2:** Impact of Radiation or Chemoradiation Therapy on the Head and Neck Microbiome.

Source	Aim	Treatment Modality (sample Size)	Bacterial identification	Observations	Main limitations
**Oral cavity**					
Saliva					
Xu et al (25)	Characterize the oral microbiota of patients undergoing CRT	CRT (n=3) for NPC; Historical controls (n=3)	16S rRNA gene amplicon pyrosequencing (Roche 454 GS-FLX)	- ↑ *Neisseria, Leptotrichia* and *Pseudomonas*, and ↓ *Streptococcus* in NPC *vs*. controls	- Limited sample size with inter-subject variability
					- Inadequately matched controls
Zhang et al (26)	Investigate the relationship between salivary function, oral microbiota, and radiation caries	RT without caries (n=12); RT, with caries (n=9) for NPC	16S rRNA gene amplicon sequencing (Applied Biosystems)	- Changes in salivary function and oral microbiota post-RT do not explain the absence of radiation caries in radiation caries-free individuals	- Study-design (cross-sectional)
Kumpitsch et al (3)	Characterize change in bacterial, fungal, and archaeal components of the salivary microbiome	CRT (n=31) for HNSCC; Control (n=11)	16S rRNA gene and ITS amplicon sequencing (Illumina MiSeq)	- ↑ fungi (e.g., *Candida*)	- Limited sample size
				- ↓ bacteria (relative ↑ *Bifidobacterium)*	- Heterogeneity of subjects (treatment and demographics)
				*-* No change in archeal composition	
Buccal mucosa					
Mougeot et al (27)	Determine changes in oral microbiome across RT treatment and characterize association with dental caries	RT (N = 31) for HNSCC	16S rRNA gene amplicon sequencing (modified Illumina MiSeq)	- Significant changes in RT-treated oral microbiome (e.g., ↑ *Streptococcus mutans*)	- Limited sample size
				- ↑ Tooth decay associated with ↓ *Abiotrophia defective*	- Inability to measure strain-level differences
				*-* Caries not associated with a difference in salivary flow	
Reyes-Gibby et al (28)	Determine changes in oral microbiota on OM onset, incidence, and severity	CT only (n=2); RT only (n=7); CRT (n=57) for HNSCC	16S rRNA gene amplicon sequencing (Illumina MiSeq)	- 86% of patients developed OM, median onset 21 days	- Limited sample size
				- ↑ Genera of normal microbiota (varying throughout the course of treatment) associated with onset and severity of OM	- Dietary changes during therapy not controlled for
Saliva and buccal mucosa					
Vesty et al (29)	Determine changes in oral microbiota on OM incidence and severity	RT (N = 19) for HNSC	16S rRNA gene and ITS amplicon sequencing (Illumina MiSeq)	- ↑ Candida, not associated with OM incidence and severity	- Limited sample size
				- GNB: some positively correlated with OM incidence; others, with OM severity	- Study design (no control group)
				- Little variation throughout the course of treatment	
Supragingival plaque					
Hu et al (30)	Study variations in oral microbiota of supragingival plaque during RT	RT (N= 8) for HNC	16S rRNA gene amplicon pyrosequencing	- Negative correlation between operational taxonomic units and radiation dose	- Possible sampling error (site-specific oral microbiome)
			(Roche 454 GS-FLX)		
Gao et al (31)	Study variations in oral microbiota of supragingival plaque before and after RT	RT (N = 3) for HNC	16S rRNA gene amplicon pyrosequencing	- ↑ RT dose results in ↓ diversity and richness of the oral microbiome.	- Limited sample size
			(Roche 454 GS-FLX)	- Gradual return to pre-RT microbiome post-RT completion	- Data analysis method (inability to detect fungi)
**Pharynx:** Oropharyngeal swab					
Hou et al (32)	Determine changes in oral microbiota on OM incidence and severity	RT alone (n=3); CRT (n=16) for NPC	16S rRNA gene amplicon sequencing (Illumina HiSeq2000)	- Genera in OM strongly correlated with genera in periodontal disease	- Limited sample size
				- *Prevotella, Fusobacterium, Treponema, and Porphyromonas* varied in abundance throughout RT: peak associated with the onset of severe OM	- Data analysis method (poor understanding of temporal dynamics)
Oliva et al (33)	Characterize oral and gut microbiome pre- and post- CRT in HPV+ patients with OPSCC	CRT (N=22) for OPSCC patients HPV+	16S RNA and shotgun metagenomic sequencing (Illumina Nextera Flex)	- ↓ number of species and ↑ relative abundance of gut-associated obligate anaerobes in oropharyngeal swabs	- Limited sample size
					- Possible unaccounted confounders (e.g., dietary habits)
**Both Oral Cavity and Pharynx**					
Zhu et al (34)	Determine changes in oral microbiota on OM incidence and severity	RT only (n=17); Induction CRT + RT (n=2); CRT + CT (n=23) for NPC; Control (n=49)	16S rRNA gene amplicon sequencing (Illumina HiSeq2000)	- ↓ bacterial diversity and ↑ *Actinobacillus, Mannheimia and Streptobacillus, and unclassified Pasteurellales and pasteurellaceae*	- Limited sample size
				associated with increased OM severity	
Lim et al (35)	Determine changes in oral microbiome and metabolomic profiles up to 24 months post-CRT	RT or CRT for OCC (n=9) and OPC (n=20)	16S rRNA gene amplicon sequencing (Illumina MiSeq)	- Most genera ↓ immediately post-treatment and most gradually return to pre-radiation levels	- Limited sample size
				- ↑ relative abundance of *Lactobacillus* and *Streptococcus* immediately post-CRT	
				- downregulation of nitric oxide metabolites post-CRT in saliva samples	
**Nose and Paranasal Sinuses:** Sinonasal swab					
Stoddard et al (36)	Describe and compare the sinonasal microbiota of rhinosinusitis after RT using culture and molecular techniques	RT (N = 22) for SN, NP, and SB cancer (9 types)	16S rRNA gene pyrosequencing (Micro GenX) *vs*. culture	- Molecular analyses are superior to culture (↑ number and diversity of microorganisms)	- Limited sample size
				- Post-RT sinusitis microbiota was similar to that of chronic sinusitis in healthy adults	- Study design (retrospective)

CRT, chemoradiotherapy; CT, chemotherapy; GNB, gram-negative bacteria; HN, head and neck; HNC, head and neck cancer; HNSCC, head and neck squamous cell carcinoma; NP, nasopharyngeal; NPC, nasopharyngeal carcinoma; ITS, GNB, gram-negative bacteria; OCC, oral cavity cancer; OM, oral mucositis; OPC, oropharyngeal cancer; OPSCC, oropharyngeal squamous cell carcinoma; rRNA, ribosomal RNA; RT, radiotherapy; SB, skull base ; SN, sinonasal. Symbols: ↑, increase; ↓, decrease.

Until the last decade, microbial research in irradiated HNC patients was entirely culture-based (3, 37). Cultivation-based analyses greatly limit the identification of microbial species, thus providing significantly less information on biodiversity and polymicrobial environments and no data on the function of the microbiome (38). Additionally, cultures are time-consuming and thus non-desirable when speedy identification is warranted (39).

Nonetheless, much of what we know about the effects of RT on head and neck microbiota, especially in the oral cavity, is due to culture-based methods. There is convincing evidence to suggest that RT modifies the composition of oral microbiota, mainly with an increase in the number of gram-negative bacteria [e.g., *Klebsiella* sp (40, 41), *Pseudomonas aeruginosa (* 41)], *Candida albicans (* 40–44 *)*, and some gram-positive bacteria, most importantly *Lactobacillus* sp (42, 43, 45). While prior research supports the association between microorganisms identified in RT-treated oral cavities of HNC patients and RT-induced toxicity (5, 45–48), the behaviors and functions of the head and neck microbiome (HNM) have yet to be fully understood as a potential explanatory mechanism (16). In a randomized clinical trial, Stokman et al. sought to assess the impact of selective microbiota elimination using topical broad-spectrum antibiotic treatment (polymyxine E, tobramycin, and amphotericin B) compared to placebo on the development of radiation-induced OM (47). The authors concluded that selective elimination is not effective in preventing the development of OM. Such findings have formed the basis for the research presented from here onwards, using culture-independent molecular analyses.

Multiple RT and CRT-induced changes in HNM have been discovered in the past decade using molecular analyses. An increased number and diversity of fungi, in addition to *Candida*, have been detected (e.g., *Peniophora, Stereum, Cladosporium*) *(* 3 *).* The total number of bacteria tends to decrease immediately after treatment and gradually return, while the relative abundance of certain species (e.g., *Streptococcus mutans*) and genera (e.g., *Bifidobacterium, Lactobacillus)* tend to increase and reflect gut-associated obligate anaerobes (3, 27, 28, 33, 35). Regarding archea, there seems to be little to no change due to RT (3). Furthermore, recent studies have investigated the impact of varying radiation doses, noting an inverse relationship between dose and microbiome diversity (31, 37). Additionally, much of recent research has characterized the HNM of patients with evidence of RT-induced tissue toxicity (e.g., OM) (28, 29, 32, 34). Other studies within the oral cavity have investigated the variation of RT-induced changes in the microbiome of supragingival plaque in HNC patients (30, 31), and possible associations with the incidence of dental caries (26, 27).

### Oral Mucositis

OM is one of the most incapacitating side effects of RT, CT, or CRT for HNC and thus one of the most extensively studied (5, 49–51). The World Health Organization’s OM Toxicity Scale ranges from grade 1 to 4: (1) soreness with or without erythema, (2) ulcers with erythema without dysphagia to solid foods, (3) ulcers with extensive erythema and dysphagia to solid foods, and (4) mucositis with complete dysphagia (5). Grades 3 and 4 are considered severe. Nearly 90% of HNC patients receiving RT develop OM, with more than two-thirds of cases classified as severe (5, 49).

In the traditional model for understanding the pathophysiology of therapy-induced OM, radiation causes tissue oxidation, DNA damage, and cell death, followed by a pro-inflammatory response causing secondary tissue damage; this leads to ulcer formation with translocation of bacteria leading to more inflammation, and subsequent healing (52, 53). However, this model should no longer form the sole basis for understanding OM pathophysiology due to the passive role it attributes to the oral microbiome, which is known to modulate the immune system (54, 55). Radiation-induced dysbiosis is thought to disrupt the immune response, which may affect the development and persistence of ulcerations (28).

In aims of bettering our understanding of OM pathophysiology, incidence, and severity, studies have described changes in oral microbiome relative abundance and diversity post-exposure to RT/CRT. Zhu et al. (34) studied the oral microbiome of 41 patients receiving RT alone or in various combinations with CT for nasopharyngeal carcinoma (NPC). They noted that a decreased bacterial diversity and an increased abundance of *Actinobacillus, Mannheimia, Streptobacillus*, and *unclassified Pasteurellales and pasteurellaceae* were associated with increased OM severity. Interestingly, Vesty et al. (29) found that an increased abundance of *Candida* was not associated with OM incidence *nor* severity, but that specific gram-negative bacteria (e.g., *Fusobacterium, Haemophilus*) were associated with OM susceptibility, and that others (e.g., *Porphyromonas* and *Tannerella*) were associated with greater OM severity. In another study (32), the peak in abundance of similar microorganisms (e.g., *Prevotella*, *Fusobacterium*, *Treponema*, and *Porphyromonas*), found in necrotizing ulcerative gingivostomatitis (53), were associated with the *onset* of severe OM. The authors in the latter study reinforced the importance of culture-independent techniques by identifying uncultivable pathogens (e.g., *Filifactor, Selenomonas, Parvimonas, Peptostreptococcus*) found in periodontal disease (32). Finally, a recently published study (28) noted an onset of OM at 21 days post-CRT and identified different genera at varying stages of OM progression: *Prevotella, Fusobacterium*, and *Streptococcus* immediately before OM appearance, and *Megasphaera* and *Cardiobacterium* immediately before the development of *severe* OM.

### Dental Plaque and Caries Formation

Dental plaque is composed of a diverse microbiota. In symbiosis (i.e. microbial homeostasis), neighboring oral tissue (e.g., teeth) remain healthy (56). Factors such as inadequate salivary flow and diet intake can modify the microbial habitat, which in combination with numerous other genetic, immunological, environmental, and behavioral factors leads to dysbiosis, which favors the growth of dental plaque and caries formation (57, 58). Dental plaque is primarily composed of Firmicutes and Proteobacteria phyla and gram-negative, anaerobic species such as *Veillonella parvula*, *Streptococcus oralis*, and *Streptococcus mutans* that favor tartar formation, dental caries, and periodontal disease (56, 58).

Several studies have sought to investigate the changes of the dental plaque microbiome in different pathological states. Hu and colleagues (30) studied the oral microbiome of supragingival plaque during RT for HNC and found a negative correlation between operational taxonomic units and radiation dose. This finding was corroborated by a study in 2015 by Gao and colleagues (31), who noted that as RT dose increased, the diversity and richness of the oral microbiome in supragingival plaque decreased during RT but gradually returned to normal post-RT completion.

Thus, RT appears to perturb plaque composition only temporarily. Whether such RT-induced changes lead to dental caries formation has previously been studied. Zhang et al. (26) compared two groups, both had NPC and underwent RT. One group had RT-induced caries (n=12), the other was caries-free (n=9). The oral microbiome was compared to determine its relationship with the presence or absence of carious lesions. These authors found that oral microbiome post-RT could not explain the absence of radiation caries in radiation caries-free individuals. Another study noted that increased tooth decay was associated with decreased *Abiotrophia defective*, a potentially protective oral gram-positive bacteria (27). Thus, compared to OM, the relative RT-induced microbiome alterations as explanatory mechanisms for dental caries occurrence seem to have less of a role or have yet to be adequately elucidated.

### Effect of Head and Neck RT on Non-Oral Cavity Subsites

Only a single study (36) investigated RT-induced microbial changes outside the oral cavity and oropharynx. In 2019, Stoddard et al. described and compared the sinonasal microbiota of rhinosinusitis post-RT using culture-based and molecular techniques (36). They collected sinonasal DNA samples of 22 patients with one of nine types of sinonasal, nasopharyngeal, and skull-based neoplasms. The authors recognized molecular analyses as superior to culture-based methods due to their capacity in detecting a greater number and diversity of microorganisms; albeit, both methods identified *Staphylococcus aureus* as the most common organism followed by *Pseudomonas aeruginosa.* They noted that the post-RT sinusitis microbiota was similar to that of CRS in healthy adults (36).

Although data on RT and the microbiome of non-oral cavity subsites is limited, several studies of the HNM without relation to RT are available; interested readers are referred to recent reviews, discussing the ear (59), nose (60), pharynx, and larynx (61, 62).

## The Head and Neck Microbiome and Response To Treatment

The microbiome also plays a key role in predicting RT efficacy. Studies from the gut microbiome suggest tumor-induced dysbiosis prior to RT and RT-induced microbiome perturbation as mechanistic explanations for reduced RT effectiveness (11, 16, 63, 64). Although severe RT-induced toxicity (e.g., severe OM) can limit treatment completion and success (64), no study aimed at determining RT effectiveness as a function of the HNM was identified.

Understanding the role of the microbiome in cancer treatment has several clinical implications. One such implication is modifying the HNM to prevent the development of severe OM (5). For example, a recent randomized controlled trial (65) of 99 patients with NPC undergoing CRT found that patients who received concomitant probiotics during CRT had a significant decrease in the incidence of severe OM compared to controls who had CRT alone (15.5% *vs*. 45.7%, respectively). Analysis with 16S rRNA gene sequencing before and after CRT indicated that the use of probiotics restored microbial diversity to that of healthy individuals. Other studies observed that a reduction in severe OM due to RT is associated with higher rates of treatment completion (64).

## Future Directions

The findings discussed in this review have uncovered several opportunities for future study of the HNM in response to RT/CRT, both from a methodological and therapeutic perspective.

The studies we present have methodological advantages over culture-based techniques: superior sensitivity, specificity, and speed in detecting microorganisms that may be responsible for causing RT-induced toxicity (39, 66). By using “sequencing by synthesis” methods that target the 16S rRNA subunit (67), studies in this review allow for identification of microorganisms at the phyla and genera levels, while one study (33) used metagenomic shotgun sequencing to capture species-level changes. Recent advancements in molecular processing have made possible the use of a multi-omic approach—metagenomics, metabolomics, metatranscriptomics, and metaproteomics (68)—to identify strain-level microorganisms and obtain additional information on how the microbiome may be related to clinical changes in the host (27, 69).

Additionally, study of the HNM in patients undergoing RT/CRT has primarily sought to characterize dysbiosis and establish a possible link with patient symptomatology, particularly in severe OM. However, limited research is available on whether RT-induced changes in the HNM are responsible for resistance to treatment and why this may occur. Nevertheless, targeted therapy is actively being investigated for HNCs resistant to treatment. One example is the emerging use of immune checkpoint inhibitors (immunotherapy drugs that prevent immune checkpoints proteins from inhibiting T cells intended to destroy cancer cells) as first-line treatment or in combination with RT/CRT (70). Moreover, bacteriophage therapy, a type of personalized medicine that spares healthy cells—unlike broad-spectrum antibiotics (18)—are now being studied in the HNM of non-cancer patients *(* 71 *).* Additionally, although not yet studied in the HNM (72), the use of microbiota transplantation from responsive donors may increase response to immunotherapy, given the established role of the microbiome in modulating immune response (73).

One important challenge in studying the oral microbiome is that the diversity of microorganisms is both individual- and site-specific. In the oral cavity, specific microorganisms preferentially reside in certain microbial loci or sub-niches (e.g., tongue, cheek, and teeth) (74). For example, the microbiome of subgingival plaque differs from that of the resident dorsal tongue, and the tongue has a different microbial diversity than the buccal mucosa (12). Such specificity also exists within a given oral cavity structure; for example, the microbiome of subgingival plaque varies according to the tooth surface (75). Hence, researchers should carefully define the intraoral site for which data is collected and analyzed.

Our review noted that most studies were focused on the oral microbiome, whereas a single study (36) investigated the impact of RT on the sinonasal microbiome. Approximately one-sixth of patients undergoing RT for NPC will experience RT-induced rhinosinusitis (76), believed to be secondary to tissue injury, inflammation, and edema to the sinonasal cilia and mucus-secreting cells (77–79). Thus, despite the frequency of tissue toxicity, the impact of RT/CRT on the sinonasal microbiome remains understudied.

Therefore, future studies should characterize the sinonasal microbiome of patients treated with RT/CRT, and prospectively evaluate patients during treatment, using molecular methods, while considering the use of a multi-omic approach to detect change over time at the strain level. Furthermore, microbiome dysbiosis identified in preliminary studies should be considered with respect to patient symptomatology and response to treatment. Although this review explored dysbiosis in relation to cancer therapy, dysbiosis due to various intrinsic and extrinsic factors is associated with cancer development through inflammation and may, in part, be caused by cancer itself (72, 80); identification of possible pathophysiological mechanisms linking this complex relationship should be a core objective of future research, allowing for the eventual proposition and assessment of therapeutic modalities (e.g., probiotics) and ultimately tailored care. Finally, future research should be conducted with adequately powered populations, as discussed by several studies (3, 25, 27–29, 31, 32, 34–36), who, for the most part, stated the need for larger sample sizes.

## Conclusions

The study of the microbiome in HNC as an explanatory mechanism and potential therapeutic avenue for overcoming the limitation of locoregional toxicity and response of RT and CRT has gained significant interest in recent years. Analysis of the HNM using molecular methods has uncovered various microorganisms not previously known using culture-based methods, providing helpful insights into RT-induced OM, dental plaque, and caries formation. Our review highlights the need for future research to (i) explore further the effect of RT and CRT using molecular techniques, on not only the oral microbiome but also on the sinonasal and oropharyngeal microbiome, in adequately powered populations; (ii) to identify if dysbiosis was accompanied by patient symptomatology and response to treatment and explore mechanisms by which these occur; and finally (iii) to propose and investigate potential modes of intervention.

## Author Contributions

IZ-O, MD, and AM contributed to conceptualization and design. All authors contributed to analysis and interpretation of data. IZ-O and AM drafted the manuscript. All authors critically revised the manuscript for important intellectual content and approved it for publication.

## Conflict of Interest

MD has received research funding from GSK, AstraZeneca, Sanofi/Regeneron, is part of the speaker’s bureau for AstraZeneca and MEDA, and is founder, owner, and president of Probionase Therapies.

The remaining authors declare that the research was conducted in the absence of any commercial or financial relationships that could be construed as a potential conflict of interest.

## Publisher’s Note

All claims expressed in this article are solely those of the authors and do not necessarily represent those of their affiliated organizations, or those of the publisher, the editors and the reviewers. Any product that may be evaluated in this article, or claim that may be made by its manufacturer, is not guaranteed or endorsed by the publisher.
